# Addressing the lack of ethnic diversity in the UK paramedic profession: a call for action

**DOI:** 10.29045/14784726.2025.6.10.1.62

**Published:** 2025-06-01

**Authors:** Amanda Rodrigues Amorim Adegboye, Ufuoma Jones, Gary Gilkes, Barbara Kozlowska, Julia M. Carroll, Tracey Rehling, Emmanuel Antwi, Kacper Sumera, Megan Parr, Candice Mansell, Rosie Kneafsey

**Affiliations:** Coventry University ORCID iD: https://orcid.org/0000-0003-2780-0350; Coventry University ORCID iD: https://orcid.org/0000-0003-2639-3773; Coventry University; West Midlands Ambulance Service University NHS Foundation Trust ORCID iD: https://orcid.org/0000-0003-1869-340X; Coventry University ORCID iD: https://orcid.org/0000-0002-3614-6883; Coventry University; Coventry University; East Midlands Ambulance Service NHS Trust; European Pre-hospital Research Network ORCID iD: https://orcid.org/0000-0002-1986-498X; Coventry University; NHS England; Coventry University ORCID iD: https://orcid.org/0000-0002-6613-5296

**Keywords:** ambulance service, diversity, education, higher education institutions, inclusion, paramedic, workforce diversity

## Abstract

Ethnic minorities are starkly underrepresented among paramedics in the UK, with their numbers significantly lower than in other allied healthcare professions. This disparity is not reflective of the diverse population the NHS serves. To achieve truly patient-centred care, the paramedic workforce must be representative of the communities it serves. This professional practice article aims to discuss the role of higher education institutions, NHS England and NHS ambulance trusts in addressing this issue.

The article examines traditional entry routes to paramedic education and highlights financial constraints and limited entry points as significant barriers for aspiring paramedics from diverse backgrounds. Furthermore, a lack of ethnic diversity in paramedic leadership positions is identified as a discouraging factor. The article then critically appraises existing initiatives aimed at promoting diversity, such as the College of Paramedics’ campaign. It acknowledges that raising awareness is a valuable step forward and advocates for a more comprehensive approach to achieve greater impact.

The article lists some of the strategies employers could adopt to foster a more inclusive workplace culture and to support the career progression of paramedics from ethnic minority backgrounds. These strategies include targeted outreach programmes, mentorship initiatives, diversity and inclusion training for all staff and revisions to promotion policies.

The article concludes by emphasising the need for a multi-pronged approach, involving collaboration between educational institutions, ambulance services, professional bodies and the government. Through implementing targeted recruitment strategies, fostering inclusive workplaces and providing career development opportunities, stakeholders can work together to build a more diverse and representative paramedic workforce, ultimately leading to improved equitable health outcomes at the individual, group and population level.

## Introduction

The lack of ethnic diversity in paramedic education and practice in the UK is a persistent and concerning issue that demands immediate attention. Despite the UK’s multicultural society, the paramedic workforce remains predominantly homogenous (College of Paramedics, n.d.). This lack of ethnic diversity not only represents a missed opportunity for inclusivity but also raises questions about the ability of the profession to provide equitable care to all communities served. Research indicates that a diverse healthcare workforce can significantly improve patient outcomes, particularly for ethnic minority communities. For example, culturally competent care has been linked to higher patient satisfaction, better adherence to treatment plans and improved overall health outcomes (NHS England, 2023a). Therefore, addressing the underrepresentation of ethnic minorities in the paramedic profession is not only a matter of social justice but also a critical component of patient safety and quality care.

This professional practice article discusses the role of higher education institutions (HEIs), NHS England and NHS ambulance trusts in addressing this issue. Existing initiatives are explored and critically appraised to demonstrate how they align with strategic plans such as the Allied Health Professions (AHP) Strategy for England (2022–2027), the NHS Workforce Race Equality Standard (NHS England, 2023b) and the new NHS Long Term Workforce Plan (NHS England, 2023a).

## The current state of ethnic diversity in the paramedic profession and education

Although the overall percentages of ethnic groups applying to HEIs are low, the paramedic profession in the UK has a particularly low representation of Black, Asian and other ethnic minority backgrounds, at around 5% (HCPC, 2021a), compared to all other AHP and Health and Care Professions Council (HCPC)-registered staff, which have a representation of 15% and 22.1%, respectively (HCPC, 2021a). This modest representation relates to a lack of role models for minority students, an understanding of the role and work patterns of paramedics, including discrimination, a feeling of separation, and racially insensitive communication (Farquharson et al., 2017).

Combined Workforce Race Equality Standard (WRES) data from all English ambulance services in 2019 show that the median proportion of non-White staff is 3.6%, ranging from 1.5% at North East Ambulance Service NHS Foundation Trust to 15.5% at London NHS Ambulance Service (College of Paramedics, 2020). Even in London the 15.5% representation of non-White staff remains below the population demographic of 18.3% of Black, Asian, Mixed or other minority ethnic population in the UK reported in the last census (Office for National Statistics, 2022), highlighting systemic barriers to inclusion.

Addressing the underrepresentation of Black, Asian, Brown, Mixed ethnic background and minoritised ethnic students in paramedic science courses is imperative across all educational levels, from undergraduate studies to advanced degrees. Current admissions practices may inadvertently perpetuate this lack of inclusivity in paramedic education (O’Sullivan et al., 2019). For instance, Higher Education Statistics Agency data from 2013/2014 reveal that only 3.4% of paramedic learners in HEIs hailed from Black or minority ethnic backgrounds (Health Education England, 2023). More recent University and Colleges Admissions Service (UCAS) data from 2023 underscore this ongoing disparity; while 13% of accepted offers to paramedic courses (n = 2055) were from non-White applicants, ethnic minorities remain underrepresented (UK Government, 2023a). By comparison, UCAS data from 2023 indicate that acceptance rates for 18-year-old applicants across all HEI courses were higher for ethnic minority groups: 50.6% for Black applicants, 53.9% for Asian applicants and 70.7% for Chinese applicants (UCAS, 2023). This stark difference highlights systemic barriers that disproportionately hinder ethnic minority students from accessing paramedic science education.

To effectively tackle this issue, promoting diversity through targeted undergraduate student recruitment initiatives is vital. Thus, comprehending the existing pathways to paramedic education and recognising the potential for HEIs to enhance diversity are crucial steps in mitigating this disparity.

## Routes to paramedic education and the role of higher education institutions

The typical route to becoming a paramedic involves completing a three- or four-year full-time BSc Hons Paramedic Science degree at a university (Weber et al., 2024b). These programmes blend theoretical knowledge with practical skills honed through placements within ambulance services. The 2023–2024 North West Ambulance Service report (2024) shows that while the Trust provides ‘earn while you learn’ schemes and apprenticeships, the dominance of the full-time degree pathway presents barriers for specific communities, particularly regarding socio-economic background. However, about 1000 staff have been trained nationally through an apprenticeship scheme in the past two years, significantly increasing the UK’s paramedic workforce (North West Ambulance Service NHS Trust, 2023), with reports from South Western Ambulance Service (2024) indicating that recruitment into the NHS ambulance service through apprenticeship is diverse and accessible to all. Financial constraints are a significant hurdle for potential paramedics from underrepresented groups, as traditional paramedic degrees typically incur tuition fees and living costs, potentially excluding talented individuals from low-income backgrounds. Furthermore, entry requirements often emphasise specific science subjects at GCSE and A-level, which may disproportionately disadvantage students from schools with limited access to resources and qualified science teachers. This perpetuates a cycle of homogeneity within the paramedic profession.

The NHS’s Long Term Workforce Plan, published in June 2023, positions apprenticeships as a key strategy to address staffing shortages within the AHPs (NHS England, 2023a). This focus holds significant promise for enhancing diversity and inclusion within the paramedic workforce. Apprenticeships, with their blend of work-based learning and off-the-job education, offer a more accessible pathway, potentially attracting a wider pool of candidates and removing financial barriers associated with traditional university degrees (The King’s Fund, 2023). Furthermore, by fostering close collaboration between NHS trusts and educational institutions, apprenticeship schemes can be tailored to address regional diversity needs and actively target underrepresented communities.

Since 2021, a degree has become mandatory for paramedic registration, although alternative routes, such as foundation degrees and top-up degree programmes, remain available (HCPC, 2021a). These programmes allow paramedics with existing FdSc/DipHE qualifications to enhance their credentials in preparation for the evolving roles in pre-hospital emergency care (HCPC, 2021b). These options may attract individuals from diverse backgrounds who possess transferable skills and a strong commitment to healthcare, ultimately supporting a more inclusive and versatile paramedic workforce.

HEIs can potentially act as a powerful vehicle for social mobility, breaking down barriers and opening doors to individuals regardless of their socio-economic background (UK Government, 2012). In 2021 the HCPC conducted a voluntary survey of professionals, collecting diversity data from 51,710 registrants across 15 health and care professions, which accounted for 18% of all HCPC registrants. The findings revealed that paramedic professionals were less likely (22%) to report having a parent with a higher qualification compared to other professions. This suggests that paramedic students may often come from backgrounds with lower levels of formal education.

The Sutton Trust (2021) report on university and social mobility shows that acquiring a university degree acts as a springboard for social mobility, offering young people from disadvantaged backgrounds a greater chance of reaching higher income and narrowing the gap between them and their peers. The report also shows that top-ranking institutions for social mobility are mainly less-selective universities in London and that they exhibit a combination of high access rates and favourable earning outcomes. Many universities in the UK have widening participation schemes. These initiatives actively target students from diverse communities, bridging the gap through targeted outreach activities, mentorship schemes and financial aid (UCAS, 2023). Some HEIs may also have a fair access scheme that includes a package of support for applicants with significant barriers to their education in addition to reduced offers (dependent on the course and current qualifications) (Department for Education, 2023). These initiatives could be targeted at areas such as paramedic sciences, which currently have an underrepresentation of minority groups.

In addition to widening access, universities need strategies to retain students and support their academic attainment. This includes adopting strategies for minority students, such as an asset-based approach. Asset-based approaches focus on what ethnic minority students can contribute to the classroom to address attainment problems (Samuelson & Litzler, 2016), with universities recognising the cultural wealth they bring. This helps develop teaching, learning and assessment, enabling learners to see themselves in the curriculum, feel a sense of belonging and understand how to succeed academically (Garoutte & McCarthy-Gilmore, 2014). The principal focus of the asset-based model is identifying and promoting strengths and resources within communities, organisations and individuals. However, the negative angles of this approach may include over-reliance on asset-based approaches and failure to address power differences among students, meaning that a balance should be considered. Frameworks for asset-based models vary but commonly avoid a deficit-based approach, encouraging the attributes and assets that individuals possess (Kretzmann & McKnight, 1993).

Integrating cultural humility and anti-discriminatory practices into the curriculum is essential for building a culturally competent workforce and reducing health disparities. This requires critical examination of healthcare through diverse lenses, as well as self-bias awareness and an understanding of how structural factors impact access (Chae et al., 2020; Solchanyk et al., 2012). By fostering inclusive learning environments and equipping future paramedics with the tools to navigate discrimination, including ensuring that the voices of paramedics, irrespective of their backgrounds, are heard in the affairs of the College of Paramedics (College of Paramedics, 2020), HEIs can ensure graduates are prepared to serve a diverse patient population effectively. The presence of paramedics from diverse backgrounds on campus is crucial in promoting diversity in the profession. Paramedics serve as role models, shattering stereotypes and demonstrating to students from similar communities that a career in paramedicine is attainable. Their lived experiences and insights enrich the learning environment, inspiring future generations to follow in their footsteps.

## The role of NHSE

Organisations such as Health Education England, now part of NHS England, have shaped healthcare education and workforce development in England. Recently, efforts have targeted increased diversity in paramedic education (NHS England, 2023a). This includes revising admission criteria to ensure equitable access for underrepresented groups, offering scholarships and financial support to encourage diversity and collaborating with universities to develop culturally sensitive curricula.

The Next Gen Lead programme aims to enhance equity, diversity, inclusion and belonging (EDIB) within AHP careers, including paramedicine, which represents only 3% of the total in the region (The Talent Foundry, 2024). To address this disparity, NHS England proposes increased recruitment from underrepresented backgrounds by HEIs (NHS England, n.d.). It is crucial to recognise that without a diverse pool of learners, there will be a lack of diversity within the paramedic workforce (College of Paramedics, 2023). Achieving this goal requires collaboration between HEIs, healthcare trusts and NHS England (Weber et al., 2024a). Besides targeted recruitment and apprenticeship schemes, there are currently no specific strategies to address the underrepresentation of paramedics from diverse backgrounds. However, the College of Paramedics (2023) suggests a need for some underrepresented communities to be encouraged to apply to paramedic education, with the encouragement involving people seeing and hearing from other paramedics who are from the same or similar backgrounds.

## The role of employers

Throughout the UK, there is a significant lack of racial diversity in organisations, particularly in leadership positions. Leadership is widely recognised as the most influential part of any organisation in shaping its culture, values and ethics (Chartered Institute of Personnel and Development, 2017). A lack of diversity in leadership positions sets a poor example for colleagues within the organisation, for potential recruits and for the local community (Regmi & Mudyarabikwa, 2020). The Workforce Race Equality Standard (WRES, 2016) reported that White shortlisted job applicants were approximately 1.6 times more likely to be appointed from shortlisting than ethnic minority shortlisted applicants and that there was a notable absence of ethnic minority senior grade leaders within Agenda for Change pay bands. The WRES (2020) update noted that ethnic minority representation in leadership roles remains significantly lower compared to their representation in the overall NHS workforce and the local communities served. This indicates a clear need for a tailored action plan considering the entire NHS-related professional lifecycle.

The role of employers, including Ambulance Service NHS Trusts, in addressing the lack of diversity in the paramedic profession and leadership positions in the UK is crucial. As of March 2022, the WRES reports that 10% of the West Midlands Ambulance Service workforce identifies as Black, Asian or minority ethnic, and less than 5% of these individuals hold leadership positions. Although people from Black, Asian and minority ethnic backgrounds are few in leadership (UK Government, 2023b), ambulance trusts are making efforts to improve representation in such roles. For example, the North West Ambulance Service NHS Trust’s (2024) 2023–2024 report shows that they are ensuring that current and future employees represent diverse groups including at board level, with management and leadership staff being representative of their communities. Similarly, the East Midlands Ambulance NHS Trust’s 2022–2023 (2023) report indicates plans to increase the representation of people from Black and minority ethnic backgrounds in leadership roles by 1% year on year. The WRES survey in 2021 revealed that among all AHPs, specifically UK paramedics, 70.7% expressed the lowest level of confidence in their trust’s fair treatment regarding career progression and promotion (College of Paramedics, n.d.). To effectively tackle this issue, employers must actively engage in allyship and implement targeted initiatives (Chartered Institute of Personnel and Development, 2017). Allyship involves individuals, particularly those in positions of influence, advocating for and supporting the career advancement of their ethnic minority colleagues. This can include mentorship schemes, where experienced leaders guide and empower aspiring Black, Asian, Brown and Mixed ethnic background paramedics, providing them with insights and opportunities for professional growth. Furthermore, organisations can establish diversity and inclusion training to foster a more inclusive workplace culture (Royall et al., 2022). These initiatives should address biases, promote awareness of cultural differences and encourage open dialogue about the challenges faced by Black, Asian, Brown and Mixed ethnic background individuals in the paramedic profession. By creating an environment that values diversity, employers can enhance the sense of belonging for ethnic minority paramedics and support their career progression. Additionally, Ambulance Service NHS Trusts should actively review and revise promotion and career advancement policies to ensure that they are equitable and unbiased (NHS England, 2024). Regularly monitoring and reporting on the representation of ethnic minority individuals by those in leadership positions can help hold organisations accountable for progress (Institute of Government and Public Policy, 2024).

Ambulance services can offer development and intervention programmes specifically designed to address the underrepresentation of ethnic minority staff in leadership positions. Table 1 provides examples of initiatives undertaken by one ambulance service located in the West Midlands, illustrating the targeted actions employers can take to promote the career progression of ethnic minority paramedics. While this table highlights important efforts, future research should explore and compare similar initiatives across different trusts and organisations to develop a more comprehensive understanding of effective strategies.

**Table 1.** Examples of initiatives to address underrepresentation in leadership.

Initiative	Description
Direct promotion of opportunities	All leadership development opportunities are directly promoted to qualified ethnic minority staff, ensuring awareness and encouraging applications.
Retention and career conversations	Employer actively engages with ethnic minority staff to explore career aspirations and identify potential roadblocks to progression.
Co-mentoring programmes	Paramedics from minority backgrounds are paired with experienced leaders from diverse backgrounds for tailored guidance and support.
Leadership development training	The ‘Valuing difference’ module addresses unconscious biases, promotes cultural awareness and equips leaders with skills for fostering inclusive workplaces.
Cultural competence framework and toolkit	Provides leaders with knowledge and skills to navigate diverse work environments effectively.
Developing potential programme	Identifies and cultivates leadership potential among ethnic minority staff through targeted support and development opportunities.
Action learning sets	Fosters a collaborative learning environment for developing and building confident ethnic minority leaders.

Source: West Midlands Ambulance Service (2024).

In addition to the targeted initiatives listed in Table 1, employers should take further steps to create a truly inclusive environment. Implementing diversity and inclusion training for all staff, not just leadership, can foster a more respectful and supportive workplace culture (Royall et al., 2022). Furthermore, employers should ensure their promotion and career advancement policies are equitable and unbiased to dismantle systemic barriers that may hinder the advancement of ethnic minority paramedics.

## Existing initiatives

Several initiatives have been launched to promote diversity within the paramedic profession and ambulance services. However, critically evaluating their effectiveness is necessary to ensure these efforts lead to lasting change. While anecdotal evidence suggests some initiatives may yield positive results (NHS England, 2023a), a systematic approach grounded in empirical research is crucial for a comprehensive understanding of their impact (Sapey et al., 2019).

This section explores existing diversity initiatives, critically assessing their strengths, limitations and potential for improvement. Shifting focus from anecdotal accounts to robust data analysis is crucial for identifying which strategies demonstrably contribute to a more inclusive paramedic workforce. The AHP Strategy for England (2022–2027) emphasises the importance of a diverse and inclusive workforce. While it offers a strategic framework, its success depends on implementation and monitoring. Key performance indicators related to diversity and inclusion should be established to track progress and hold institutions accountable.

Reports from North Central London Clinical Commissioning Group (2022), Norfolk and Norwich University Hospitals Foundation NHS Trust (2024) and WRES (2024) indicate that the NHS WRES aims to support organisations to close the gaps in work experience between White and ethnic minority staff. The NHS WRES has the potential to drive change. However, it must be rigorously enforced to ensure compliance, meaningful progress and impact rather than the completion of actions that should be measured and reported on (Stubbs et al., 2024; WRES, 2024). The WRES should be applied to paramedic education institutions, ambulance services and related healthcare professions.

The new NHS England (2023a) Long Term Workforce Plan must prioritise diversity and inclusion as core values. This plan should include clear directives for increasing ethnic diversity in the paramedic profession and ambulance services, with specific targets and measurable outcomes. Financial incentives for institutions that meet diversity targets could be a powerful motivator for change.

The College of Paramedics launched a campaign to promote ethnic diversity in the profession (College of Paramedics, 2023). The campaign’s primary strength is raising awareness of the underrepresentation of ethnic minorities in the paramedic profession. This campaign can stimulate discussion and encourage action from stakeholders. Demonstrating the College’s commitment to diversity can potentially inspire young people from ethnic minority backgrounds to consider a career in paramedicine. However, its long-term impact may be limited without addressing systemic issues or lacking a concrete action plan. Building strong partnerships with ambulance services, universities and government bodies can significantly amplify the campaign’s impact. Additionally, regular data collection and analysis are crucial to gauge the campaign’s effectiveness and to identify areas for improvement.

While there have been some initiatives to widen participation, these initiatives are based on deficit-based approaches focusing on the limitations of students instead of their strengths (Samuelson & Litzler, 2016). Deficit-based approaches emphasise what ethnic minority students lack, including institutional barriers leading to poor attainment. Therefore, focusing on minority students’ assets fosters attainment and retention.

## Conclusion

The lack of ethnic diversity in the UK paramedic profession, paramedic education and ambulance service is an issue that compromises patient safety and exacerbates health disparities. Studies have shown that patients from ethnic minority backgrounds often experience poorer health outcomes and lower satisfaction with care when treated by healthcare providers who lack cultural competence (Raleigh, 2023). For instance, Ameneshoa (2022) argues that ethnic minority patients are less likely to receive timely and appropriate care in emergency situations, leading to higher rates of complications and mortality. Therefore, increasing diversity within the paramedic workforce is crucial for improving patient outcomes and ensuring equitable healthcare for all. Additionally, there is a need for future research comparing initiatives across trusts and organisations, which could inform best practices for addressing underrepresentation.

### Recommendations

NHS England, HEIs and employers should prioritise partnerships with community organisations and schools to inspire young individuals from diverse backgrounds to consider careers in paramedic sciences. Initiatives like mentorship programmes and outreach efforts can make a significant difference in breaking down the barriers that deter minorities from pursuing careers in this field.

While some traditional routes to paramedic education exist, barriers like financial constraints and limited diverse entry points hinder accessibility. HEIs have a responsibility to champion inclusivity by widening participation schemes, diversifying their curriculum and faculty and exploring alternative entry routes. By collaborating with ambulance services and stakeholders, HEIs can play a pivotal role in building a more diverse and representative paramedic workforce, ultimately better serving the needs of a diverse patient population.

Existing initiatives, such as the AHP Strategy for England, the NHS WRES and the new NHS Long Term Workforce Plan, provide roadmaps for change. However, their success hinges on effective implementation, monitoring and enforcement. To create a truly inclusive paramedic profession and ambulance service, these initiatives must align with broader healthcare workforce strategies and reflect a commitment to diversity and equity in patient care. The time for action is now, and we must hold ourselves accountable for making the necessary changes to ensure the paramedic profession mirrors the diverse tapestry of the United Kingdom. Table 2 summarises the key recommendations.

**Table 2.** Key recommendations for increasing ethnic diversity in the paramedic profession.

Recommendation	Potential impact/implications
NHS England, HEIs and employers partner with communities: prioritise partnerships with community organisations and schools to inspire diverse youth towards paramedic careers.	Increased awareness of paramedic careers among underrepresented groups. More diverse applicant pool for paramedic programmes. Stronger connection between the paramedic profession and the communities it serves.
HEIs champion inclusivity: widen participation schemes, diversify curriculum and lecturers and explore alternative entry routes.	Improved accessibility of paramedic education for diverse applicants. Educational programmes that better reflect and value diverse perspectives. Increased opportunities for individuals with transferable skills to enter the profession.
Effective implementation of existing initiatives: focus on effective implementation, monitoring and enforcement of AHP Strategy for England, NHS Workforce Race Equality Standard and NHS Long Term Workforce Plan.	Increased success in achieving diversity goals outlined in existing strategies. Improved accountability for achieving a more inclusive paramedic workforce. Alignment with broader NHS workforce diversity initiatives for a systemic approach.

## Author contributions

All authors provided substantial contributions to the manuscript. Their active involvement extended to reviewing and revising the manuscript critically, improving its clarity and quality. ARAA acts as the guarantor for this article.

## Conflict of interest

None declared.

## Ethics

Not required.

## Funding

None.
